# Patients understanding, perceptions and experiences of head and neck lymphoedema management following treatment for head and neck cancer: a qualitative study

**DOI:** 10.1007/s00520-025-09668-3

**Published:** 2025-06-25

**Authors:** Lauren J. Mullan, Nicole E. Blackburn, Jackie Gracey, Lynn Dunwoody, Jill Lorimer, Cherith J. Semple

**Affiliations:** 1 School of Nursing, Institute of Nursing and Health Research, Ulster University, Belfast, UK; 2 School of Health Sciences, Institute of Nursing and Health Research, Ulster University, Londonderry, UK; 3 School of Psychology, Faculty of Life and Health Sciences, Ulster University, Londonderry, UK; 4 Physiotherapy Department, Cancer Centre, Belfast Health and Social Care Trust, Belfast, UK; 5 Cancer Services, South Eastern Health and Social Care Trust, Belfast, UK

**Keywords:** Head and neck cancer, Lymphoedema, Self-management, Survivorship, Qualitative

## Abstract

**Purpose:**

Head and neck lymphoedema (HNL) is a common, unintended chronic consequence following head and neck cancer (HNC) and its treatment. Due to the chronicity of HNL, it is important to explore how patients can engage in HNL self-management. This study aims to explore patients understanding, perceptions and experiences of HNL management to promote the role of self-management and adherence to HNL interventions following completion of HNC treatment.

**Methods:**

Fourteen remote, one-to-one semi-structured interviews were conducted with HNC survivors with HNL. Reflexive thematic analysis was employed to develop key themes using an inductive approach.

**Results:**

Two main themes were established: (1) “maximising patients’ competency to self-manage HNL” and (2) “Adherence to HNL self-management: What makes a difference ?”. Data demonstrated the importance of early, clear and tailored information on HNL as a treatment consequence. Patients with HNL relied on and benefited from positive reassurance and open access to specialist HNL therapists. This promoted motivation and coping strategies to overcome the range of barriers associated with HNL self-management.

**Conclusion:**

This study identifies key barriers diminishing patients’ motivation and competency in HNL self-management, including lack of understanding, appropriate and timely tailored education, and HNL not being a patient priority. This research highlights the need to raise awareness of HNL and its chronicity, to improve the biopsychosocial burden placed on patients and promote motivation for self-management. The development of tailored HNL educational resources, grounded by the patient perspective is required to promote HNL management and improve patient’s adherence to self-management techniques.

## Introduction

Patients who have completed treatment for head and neck cancer (HNC) can experience extensive detrimental changes to both their functional and psychosocial well-being [1, 2]. These adverse treatment-related side effects may consist of dysphagia, pain, xerostomia, mucositis, nausea, dysgeusia, speech difficulties, and lymphoedema [3–6]. Head and neck lymphoedema (HNL) is reported as a common chronic consequence of HNC treatment; however, it is often under-recognised and under-treated [7, 8]. This poses a critical issue to address among this cancer population as the prevalence of HNL can be as high as 90% in patients who have completed treatment for HNC [9, 10].

HNL often can present as a result of the lymphatic system being damaged, which in turn leads to an abnormal accumulation of lymph fluid residing in the interstitial spaces of the head, face and neck [11, 12]. The nature of this accumulation of lymph fluid can lead to patients experiencing tightness and heaviness of the skin, restricted range of motion of the head and neck, swelling, changes in voice, swallowing, speech articulation and breathing difficulties [9, 11, 13]. These detrimental functional impacts have the potential to negatively influence individuals’ emotional wellbeing and overall satisfaction with their health-related quality of life (HRQOL), subsequently restricting daily life activities [10, 14]. A recent study using patient interviews evidenced this by identifying that changes in appearance and functional impairments such as difficulties in driving as a consequence of HNL, negatively impacted patients overall HRQOL [10].

Our systematic review indicated that HNL interventions to effectively manage the chronic consequences of HNL, show some promise but are still very much in their infancy [15]. The current body of literature emphasises that care for HNL is varied globally, with no single modality treatment, referral process or standardised clinical pathway [16]. The recent systematic review also highlighted a vast degree of heterogeneity across studies, involving HNL interventions, study designs and outcome measures, demonstrating the difficulty in determining effective components of HNL interventions [15].

With the growing incidence of HNC [17] and treatment advancements [18], there are increased numbers of HNC survivors living longer with the associated treatment-related side effects [19], which can require lifestyle adaptations and changes [20, 21]. Given the chronicity and impact of HNL, anatomical uniqueness and number of appointments that patients with HNC are required to attend, the role of self-management has been promoted within HNL management [22–24]. Consequently, healthcare providers approach to the management of chronic conditions such as HNL have shifted away from the more traditional professional-led approach to patients playing an important role through self-management and individualised care [20, 24, 25]. This shift in approach is deemed as necessary and expected to optimise health outcomes, accelerate recovery, mitigate long-term disability across the cancer trajectory and possibly improve survival [20, 25].

Despite acknowledgement of the biopsychosocial challenges associated with HNL [26] and the important role that self-management can play, our systematic review indicated a dearth of information and lack of evidence in the best way to successfully embed self-management into HNL interventions [15, 22, 27]. Furthermore, adherence to self-management strategies was reported as being poor overall, especially within the home-based setting [15, 28]. This evidently warrants a need to achieve a more in-depth understanding of what promotes patients long term behaviour change to improve adherence and motivation to HNL self-management techniques, which this study seeks to address [15, 23, 29].

This study aimed to explore patients understanding, perceptions and experiences of HNL management to promote the role of self-management and adherence to HNL interventions following completion of treatment for HNC. The objectives of the study included:

- Explore patients understanding of HNL management.
- Explore the perspectives and experience of patients on self-managing HNL.
- Explore and identify key barriers and facilitators surrounding HNL self-management to promote adherence to HNL interventions.

## Methods

A qualitative research design consisting of one-to-one semi-structured interviews were conducted between June and October 2024. This methodology was chosen as it can provide rich descriptive content from the perspectives of patients regarding their experiences and is often associated with planning of healthcare interventions [6, 24]. The study followed the Consolidated Criteria for Reporting Qualitative Research (COREQ) guidelines [30].

### Sample characteristics

A purposive sample incorporating patients who had previously completed treatment for HNC, with HNL and either receiving or had previously received treatment for HNL participated in this study. Participant eligibility is presented in Table 1, as established by the research team. A local collaborator was designated in each of the three participating health and social care trusts in Northern Ireland, consisting of one HNC Clinical Nurse Specialist (CNS), and two advanced clinical specialist lymphoedema physiotherapists in HNL. Local collaborators identified participants who met the eligibility criteria from follow-up review appointments. The local collaborators used the study’s inclusion and exclusion criteria to ensure all eligible participants were provided with the opportunity to participate, therefore minimising selection bias. A timeframe of one-month post-treatment was selected, to ensure no eligible participants were missed. Of note, no screening tools nor objective assessment were used as inclusion criteria. Instead, the inclusion was broad and based on patients self-reporting of HNL symptoms, as HNL is considered as under-recognised and under-treated, with no agreed process for referral or screening tool [7, 10].

**Table 1:** Study inclusion and exclusion criteria

Inclusion criteria	Exclusion criteria
• Aged 18 and over • Previous diagnosis of HNC • Treatment for HNC with curative intent completed (at least 1-month post-surgery or post-radiotherapy) • Experiencing symptoms of HNL based on patients self-reporting (no current standard objective assessment routinely performed in participating sites) • Symptoms were not restrictive and could include functional impairments: speech, voice, eating, swallowing, trismus, alimentation and range of motion in neck, shoulder and jaw. HRQOL domains: effects on emotional well-being, physical well-being, social engagement, body image issues, sexuality, loneliness, pain and quality of sleep • Able to provide informed consent • Able to speak and understand English	• Treatment being received for disease recurrence • Palliative or end of life treatment intent • Treatment for HNC not completed • Dementia or cognitive impairment

Consent was gained for the lead author (LM) to contact those participants who identified interest in participation. Thereafter, a suitable time and location was arranged for each interview. Participants were provided with the option for either a face-to-face interview when attending a review appointment, remote via the online platform MS Teams or through telephone. Written informed consent was obtained for all participants. Autonomy was ensured in this process through participants being presented with an information sheet outlining the study details, emphasising that participation was voluntary and would not have any effect on their healthcare treatment.

### Ethical approval

Ethical approval was granted for the study (OREC: 23/NI/0096), alongside research governance for the three participating health and social care trusts in Northern Ireland. A distress protocol was developed to ensure that relevant signposts for support were provided to participants due to the sensitive nature of this topic; however, this was not required. Reflective notes were made after interviews and transcription by the first author (LM) to ensure transparency and promote best practice within this qualitative research study. Regular team meetings took place to ensure sufficient content was being elicited from interviews to address the aims and objectives of this study. The standards for Consolidated Criteria for Reporting Qualitative Research (COREQ) guidelines was utilised [30].

### Data collection

**Fig. 1:**
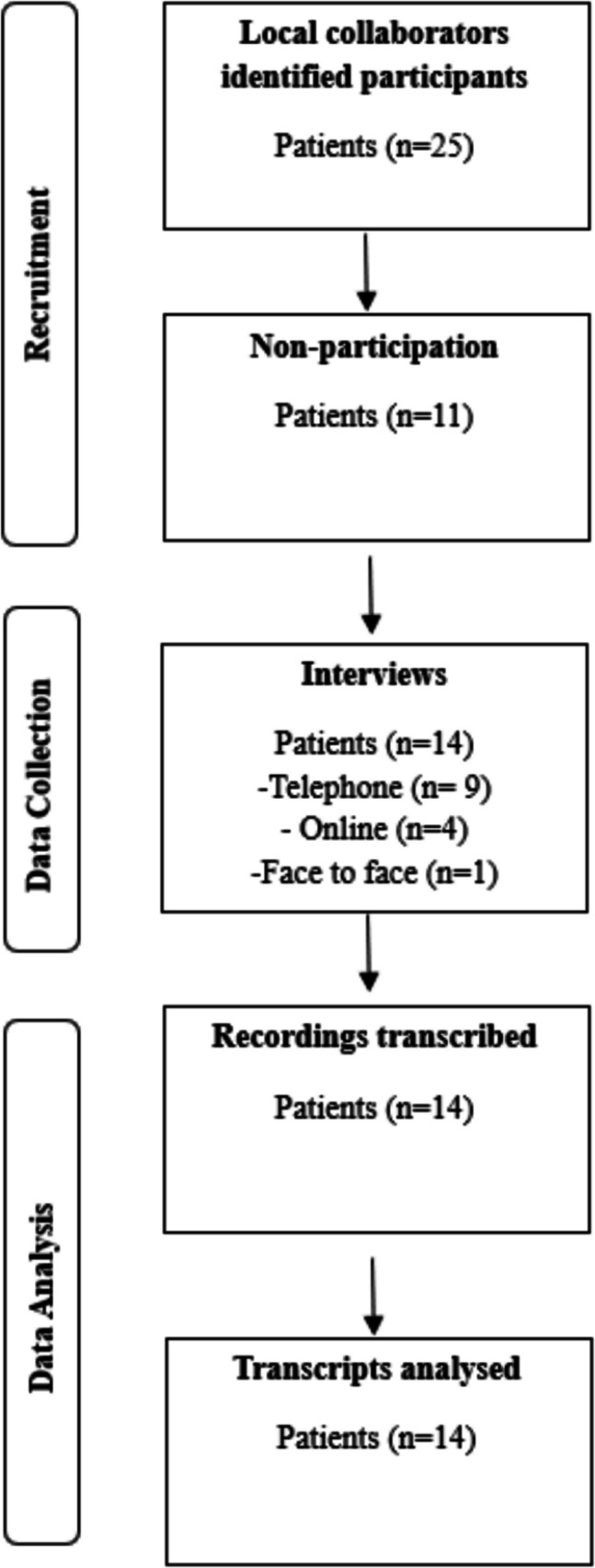
Flow diagram of data collection process

A topic guide was initially developed by lead author (LM), informed by both the existing body of literature and then refined by expert knowledge of the research study team (CS, NB, LD) and patient and public involvement (PPI) (Appendix 1). Two pilot interviews were carried out prior to the study interviews, which enabled the topic guide to be further iteratively modified. An example of this was, at the beginning of the interviews rephrasing questions to acknowledge the array of treatment-related side effects of HNC but ensuring participants understood the focus was on HNL. Based on PPI input, a decision was reached to provide a lay definition for HNL at the start of each interview, to ensure participants and the researcher had a common understanding of the phenomena of interest. Interviews were conducted using either telephone, face-to-face or online via Microsoft Teams (Fig. 1). Recent research has indicated that the use of telephone interviews does not restrict the ability to collect rich qualitative data from this study population [1, 31]. A total of 14 interviews were completed. Interviews were completed when no new major information, codes or theme were emerging from the data, ensuring comprehensive coverage of the phenomenon under study, in keeping with the values and assumptions of reflective thematic analysis (Braun and Clarke, (2021). The first author in this study (LM), a registered therapeutic radiographer and PhD researcher completed online qualitative data collection and analysis training prior to the start of the interview process and did not have direct involvement with the care provided to this participant group. Other members of the research study team (CS, NB, LD) are expert qualitative researchers. Throughout the interview process the topic guide for this study was adapted and evolved in response to transcripts being reflected on by (LM) and transcripts reviewed by (CS and NB). This coincided with best practice guidelines emphasised by Braun and Clark within qualitative research methods [32].

### Data analysis

Initially audio recordings were transcribed verbatim by either first author (LM) or expert transcriber, with study collaboration agreement. Unidentifiable transcripts were imported to NVIVO 15. Braun and Clark’s [33] six step approach to reflexive thematic analysis was followed. Familiarisation with the data was performed through reading, re-reading and making reflective notes on each transcript before any coding took place by first author (LM), to ensure rigour and reliability [33]. Transcripts were additionally checked by the research team (CS and NB) to provide validity and credibility to data analysis. The process of initial coding was conducted using an inductive approach by the first author (LM) and reviewed by team member (CS). Categories were agreed with the research team (CS and NB) to ensure they aligned with the overall study aim and objectives. Initial themes were then identified collectively by the research team (LM, CS, NB). All themes were discussed and refined to ensure that participants true meanings and interpretations were accurately presented.

## Results

Across three healthcare trusts 25 potential participants were identified by local collaborators; however, nine participants did not respond to the initial email invitation, one participant did not meet the eligibility criteria, and a further patient did not attend planned interview. In total, 11 males and three females (*n* = 14) consented to a one-to-one, semi-structured interview. Interviews were audio-recorded and varied in duration from 29 to 58 min, with an average time of 40.06 min. The participant characteristics can be viewed in Table 2. All participants age ranged from 40 to 75 years, with the most predominate tumour location being the oral cavity (*n* = 13, 93%). Most participants (*n* = 11, 79%) received a combination of surgery and radiotherapy (RT).

**Table 2:** Patient characteristics (*n* = 14)

Patient ID	Gender	Age range (years)	Tumour site	Treatment	Employment	Educational status
1	Male	66–70	Oral cavity	Surgery and RT^1^	Retired	Primary
2	Male	61–65	Oral cavity	Surgery and RT	Retired	Secondary
3	Male	61–65	Oral cavity	Surgery, RT and CT^2^	Retired	Higher
4	Female	71–75	Oral cavity	Surgery and RT	Retired	Secondary
5	Male	51–55	Oral cavity	Surgery, RT and CT	Employed	Secondary
6	Male	66–70	Oral cavity	Surgery and RT	Retired	Secondary
7	Male	51–55	Oral cavity	RT and CT	Employed	Secondary
8	Male	66–70	Oral cavity	Surgery, RT and CT	Retired	Higher
9	Female	41–45	Salivary gland	Surgery and RT	Employed	Higher
10	Female	51–55	Oral cavity	Surgery, RT and CT	Employed	Secondary
11	Male	71–75	Oral cavity	Surgery, RT and CT	Employed	Primary
12	Male	55–60	Oral cavity	RT and CT	Self-employed	Higher
13	Male	66–70	Oral cavity	RT and CT	Retired	Higher
14	Male	61–65	Oral cavity	Surgery, RT and CT	Retired	Secondary

^1^*RT* Radiotherapy, ^2^*CT* chemotherapy. NB: All participants were at least 1-month following completion of multimodality treatment

The data demonstrated that HNL appeared to have an impact on functional challenges and altered appearance, both of which had a negative effect on psychosocial wellbeing. The findings will be reported as two main themes: (1) “Maximising patients’ competency to self-manage HNL”, (2) “Adherence to HNL self-management: What makes a difference?”.

### Theme 1: Maximising patients’ competency to self-management

Participants highlighted the importance of HNL self-management to achieve the direct benefits enhancing everyday quality of life. This consequently mitigated the challenging HNL effects experienced on speech, swallow, appearance and range of motion to neck and shoulders. Maximising competency to self-manage HNL will be reported as two subthemes: (1) “Receiving HNL education” and (2) “Importance of reassurance and support from professionals and family members”.

#### Subtheme 1.1: Receiving HNL education

Most participants at the end of treatment, prior to their HNL being diagnosed by a professional had very little knowledge surrounding what HNL was, and how to manage it. Many participants expressed a desire for very basic and clear information on the pathophysiology and management of HNL early in their cancer trajectory, avoiding medical jargon and information overload. At the end of treatment, when the side-effect was most prevalent and troublesome, participants identified a need for further, more detailed information on HNL as a treatment consequence with instructional materials on intervention management. Many participants indicated a preference for this detailed, tailored information to be received at face-to-face appointments; however, some expressed positive benefits of other strategies to augment and promote recall of instructional information such as posters, tailored diagrams, leaflets and videos.

“Em? I [..] I think there's different timepoints that you could target and because I think people tend to get overwhelmed and only look at where they are at the time. I think sometimes maybe a leaflet for, you know [..] basic, for the immediate aftermath of surgery. Then or, you know a section for your, once once, once you’re through a certain part of the treatment and so on? Maybe when you're discharged by the surgeons and then a sort of a section for continuing on that, you know, a, a, after all the other treatments are finished [..] receiving more information when you’re ready.” (Patient 8).

Not having the knowledge or understanding of HNL caused anxiety, with many participants fearing their cancer had returned. Most participants did not anticipate the chronicity of HNL, nor the need to manage it long-term. A few participants raised concerns that they received conflicting information about the chronicity of HNL, creating confusion and at times fear of whether it was something worse, such as cancer recurrence.

“I don’t know if I’d been informed about lymphoedema being a potential side effect. I don’t remember being told anything about it. It was only when it manifested and em I thought ok and asked about it and they said well em you know, hopefully it will go away but it might not go away. I wasn’t expecting it or didn’t know about it so I got the symptoms and wondered what, I thought what the hell? You know, this is swelling or is it something else and because it is similar to my initial symptoms of cancer […] I suppose you’re, you do go right is there something wrong here. Is it coming back?” (Patient 12).

To promote engagement with HNL self-management patients not only required knowledge of this side-effect, but importantly the “tools” and skills required to manage the condition. Participants reported on an array of “tools” and skills, with physical exercise commonly highlighted as beneficial, alongside the helpful nature of an elevated sleeping position, manual lymphatic drainage (MLD), compression and breathing exercises. Most patients identified the importance of tailored instructional information, with one participant stating that this made them feel that they mattered and were seen as an individual.

“I think having someone who sees you as an individual and therefore even though the handouts were preprinted, they were then sort of then personalised to my situation. Having it written down and personalised or tailored to me was really helpful [..] it just felt like it was for me. And it improves the connection which means the patient is more likely to keep going with the treatment as they can see it being relevant to them and specific to them.” (Patient 3).

#### Subtheme 1.2: Importance of reassurance and support from professionals and family members

A fundamental aspect of maximising participants’ competency was reassurance provided by both HCPs and family members. Some family members provided reassurance by accompanying patients to their HNL appointments, proved purposeful for recall of information provided by HCPs, subsequently promoting adherence to HNL interventions. Furthermore, family members frequently provided positive affirmation, with a focus on minimising the perception of altered appearance caused by HNL. That said, some participants reflected on not wanting to communicate with their family about their HNL as they did not want to burden them further, after their already worrisome experience throughout HNC treatment. Most participants referred to the support from family members as predominately emotional, rather than physically engaging in the management of HNL. To a lesser extent, some participants described how their family members would have reminded them to carry out their exercises. One participant commented that their family member could tell if they had not completed their exercises because of the effect it had on their voice first thing in the morning.

“I have a wonderful family and they never changed the way they saw me. As far as I could tell, if they did they hid it extremely well. They were pleased for me when I lost the fluid, but they never saw it as a negative. And that was reassuring, that meant that my own body image was very positive because their image of me was positive.” (Patient 3).

Most participants reported that due to their initial lack of understanding towards HNL, they could not competently carry out self-management without necessary instructional support and reassurance from HCPs. The data demonstrated that it was more often a trained specialist HNL professional who proved crucial in providing detailed information, instructional support, alongside realistic and objective reassurance.

“The healthcare specialist, they you know have the experience of it. They know all about it so they are you know if your car breaks down you don’t take it to a bakery you take it to a car mechanic [..] And all the different outcomes from every patient and circumstances is different and the specialist is aware of that and can make the best decisions and explain things better [..] showing you the images or exercises I mean is the best way to go” (Patient 2).

Many participants noted the importance of having contact details and open access to a HNL specialist, especially when concerns arose with HNL self-management techniques. Open access to a HNL specialist proved extremely reassuring, often providing patients with confidence to adjust their self-management techniques. Additionally, a few participants identified that this communication provided them with a sense of control to progress and adhere to their HNL self-management programme.

“And the other thing that was really helpful was for a period of time, I was given an open access. So I could phone up and say I have a question or whatever […] the reassurance that you could do that and that you would then be seen by somebody fairly quickly was really helpful because with a lot of patients it’s about, for me it’s a lot about having the confidence and if you know you’ve got somebody who can call on, you’ll do things that otherwise you might not have done.” (Patient 3).

### Theme 2: Adherence to HNL self-management: what makes a difference?

One of the main issues reported with self-management was adherence to the various components of treatment. Most participants were seen by a specialist lymphoedema therapist and advised on skin care, exercise, compression and MLD. Adherence to HNL will be discussed under two subheadings: (1) “Physical barriers”, (2) “Factors influencing motivation and adherence”.

#### Subtheme 2.1: Physical barriers

Most participants reported that whilst they did experience benefits from wearing compression garments, it was often not easily tolerated and uncomfortable, hindering adherence as advocated by the specialist. Additionally, compression garments were regarded as unsightly, and patients certainly did not wish to wear them outside of their home.

“It was just really odd so it was to be honest because em it felt a bit like wearing a toilet seat on my head? And then, I did, I did try wearing it a couple of time, because you know, when I was watching TV or something like that? And although I do think it worked em it maybe just wasn't entirely practical either”. (Patient 9).

A minority of participants indicated that pain acted as a physical barrier to carrying out many HNL self-management techniques, especially early post-treatment. This, combined with a lack of clarity as to what was safe and how techniques could be adjusted, impacted on participants ability to carry out HNL self-management, exacerbated by fear of causing further pain or “damage”.

“I think one of the barriers is it is uncomfortable the first few times that you do it. Yeah, the the discomfort is probably a barrier […] I think from my own personal view, the biggest challenge I had was, umm, getting the balance between pressure and having dry skin on your neck. So not wanting to press too firmly and irritate the skin, but at the same time get enough pressure to move the fluid.” (Patient 3).

Finally, a few participants described feeling extremely fatigued at the end of HNC treatment, which hindered their ability to carry out exercises and MLD, especially as this took considerable time and effort when commencing HNL self-management.

#### Subtheme 2.2: Factors influencing motivation and adherence

Participants were conscious that both visualising and experiencing functional improvements were important in promoting motivation for self-management. This, in turn, encouraged adherence to the home-based programme advocated by the HNL specialist. Strategies facilitating this were objective measurements taken by professionals identifying a reduction in HNL, improved visual appearance to include before and after HNL intervention photographs and greater comfort wearing their clothing, such as a shirt. Conversely, some participants did not see an immediate improvement which reduced patients’ motivation to continue with the advocated HNL self-management techniques. Also, some participants stated that initially their self-management routines took a considerable time to perform because of lack of familiarity with their home-based programmes. Other participants described life was busy with other commitments taking priority.

“She had to get this little device, she was able to see the levels of water fluid or retention in those areas [..] she had said you know like that eh you know there was very little difference between the side of my face that was affected by it and the side that wasn’t you know so that was always really encouraging.” (Patient 9).

Accepting the chronicity of HNL as their “new norm” and subsequently fitting self-management techniques into daily life, promoted adherence to HNL intervention techniques. A few participants identified that it was the responsibility of the patients to take control and find a way to fit their self-management into their routine. For some participants, they perceived the effects of their HNL were not particularly severe, thus lessened motivation to engage in, and adhere to HNL self-management advice.

“Well, I make time anyway when I’m driving or whatever, you know? As I say when I’m stopped at traffic lights. You know I’ll just massage my neck for a minute you know. You know, so it’s routine now. And eh you need to accept that and get it into your head.” (Patient 13).

To a lesser extent, a few patients stated that having a poor cancer prognosis impacted their motivation, with the perception that they might not be alive long enough to gain the benefits from HNL self-management.

## Discussion

The findings from this study highlight that more often patients have a poor understanding of HNL, with a desire to be better informed regarding causation and management, alongside a need to receive positive reassurance from specialist HNL therapists to motivate and encourage adherence to self-management strategies. This study also clearly depicts that with HNL education and management there is no “one size fits all”; albeit tailored, non-generic information and guidance encourages engagement and fosters motivation with self-management techniques. Family members have a supportive role in HNL management, which pivots on provision of emotional support, but infrequently physical support [15, 20].

This study emphasises the importance of person-centred and tailored information to enable understanding of HNL as a post-treatment effect and to promote engagement with HNL self-management. It was clear from our study that participants desired basic information, without medical jargon on the cause and effects of HNL early in the cancer trajectory, to facilitate understanding when HNL becomes apparent, post-treatment [35]. Without a basic understanding of HNL, anxiety can be evoked, for example, patients correlate onset of submental swelling and other associated functional effects with HNC recurrence [20, 26]. Previous studies also state that without early and clear education on HNL from a specialist HCP, anxiety and distress concerning fear of recurrence can present in the survivorship phase [35, 36]. There is an important need for such brief, factual, honest and clear information to be provided either pre-treatment or during treatment to enable realistic expectations [11]. This is vital as 90% of patients following completion of HNC treatment are likely to experience HNL [5, 11].

The role of education is a fundamental component in promoting compliance, engagement and motivation with self-management in chronic conditions [37]. Patients may require different levels of skills training and support to enable them to maximise their individual ability to competently carry out self-management techniques within their daily lives [38, 39]. When individuals do not possess the knowledge or skills surrounding how to self-manage, there is greater risk of poor adherence and lack of engagement in self-management strategies [38, 39]. Other leading researchers within this field have similarly reported that patients require sufficient awareness and knowledge, to confidently manage HNL effectively [29, 40]. Predominantly the HNC population preside from lower socioeconomic backgrounds, associated with poorer health literacy [41, 42]. This is important as patients with HNC, akin to other patient populations with chronic illness need, to not only understand the information provided but be able to access and use it to effectively self-manage [41]. To achieve this, patients need to be presented with relevant, appropriate and credible information as clearly depicted by participants within this study [42].

This study indicated support for tailored information delivered via various modes, suggesting there was no “one size fits all” approach regarding an HNL educational resource. Despite this, there was commonality in the need for personalised tailored information to promote adherence and motivation for HNL self-management [13]. Furthermore, HNC patients can feel overwhelmed during treatment with the volume of information received, vast array of side effects and the number of appointments, often resulting in difficulty with information recall [35, 36]. Thus, the timing of HNL information is crucial. There was no clear consensus as to the most appropriate time and approach for patient education to promote HNL self-management. Participants in this study suggested receiving information before treatment; however, most commented it would be better to receive earlier brief information but more in-depth detailed information later in the post-treatment phase. This variation indicates that HNL education to promote self-management should not be a singular event but rather multiple timepoints may be required. Furthermore, there has been no evident consensus on a particular timepoint within the treatment trajectory for chronic illness on when best to provide self-management information, depicting individual variation [43].

Participants described the presence of positive reassurance from specialist HCPs as an important factor in maximising competency with HNL self-management, as without it they felt less confident in performing and adjusting techniques. Within the HNC population self-efficacy and reassurance can be low, emphasising the importance of positive affirmation to promote engagement and adherence [44]. This coincides with previous HNL studies as adherence rates to home-based strategies were poor due to a lack of motivation and confidence [9, 27]. Self-efficacy has been identified as an important psychological factor assisting individuals to navigate self-managing chronic conditions [40]. Considering the perceived importance of positive reassurance from specialist HCPs, HNL patients may benefit from the integration of an individualised resource, objective affirmation and goal setting to improve adherence to self-management techniques and promote long term behaviour change [27, 40]. Furthermore, this could help mitigate the demands placed on HCPs dealing with increased appointments and hospital admissions as a consequence of HNL [7, 19].

Patients also reported the importance of “open access” to a specialist HCP to provide necessary guidance and affirmation when perceived obstacles or queries arose in relation to their HNL and self-management. This model of HNL management, with accessible open lines of communication, delivered by trusted HCPs, has been reported as one of patients’ top priorities following HNC treatment [26, 29]. Open lines of communication with a HNL specialist can be beneficial, as this cohort of patients can feel overwhelmed with an array of side-effects after treatment, with lack of confidence in applying and adjusting self-management techniques without support [26, 29]. Information and advice provided by HNL specialists’ enhanced trustworthiness, positively influencing motivation and confidence to engage and continue with HNL self-management. Nonetheless, challenges exist with the provision of this “open access”, with contemporary literature depicting that HNL specialists are often a finite resource [9, 38].

As well as the important role that HCPs can play, the relationships between patients and their family members are emphasised as being fundamental in competently managing chronic conditions such as HNL [27]. Family members play an influential role in providing emotional support, with some reminding participants to carry out their self-management as well as motivating through positive reassurance. Family member involvement should be encouraged, as greater levels of family support were associated with improved adherence and control over self-management and may have the potential to reduce the demand on professional therapists [1, 20, 43, 44].

### Study strength and limitations

This study provides unique insights on patients’ educational and support needs, plus barriers and facilitators of HNL self-management, both of which can inform and facilitate the development of HNL interventions and promote adherence of self-management techniques. The study sample had more of a homogenous population of oral cavity patients that received a combination of surgery and radiotherapy, and therefore, the results cannot confidently be generalisable to other HNC sub-sites such as oropharynx or larynx. The recruitment of participants was low (*n* = 14/25; 56%), potentially linked to a lack of understanding of HNL following reading the study invitation and participant information sheet, and therefore uncertainty surrounding eligibility.

### Clinical implications

Despite HNL interventions showing promise, this field of research is still very much in its infancy. There is an imperative role that self-management must plays given the chronicity of HNL and the lack of a specialist HNL workforce [34, 35]. Given poor or partial adherence to HNL home-based interventions, the patient voice and perspective is a vital component that must be integrated during the planning and development of tailored HNL self-management interventions to enhance engagement, adherence and improve efficacy [10]. Thus, this study provides the patient perspective demonstrating an evident need to provide early and clear information to promote understanding and minimise distress, with more detailed instructional information to maximise competency in HNL self-management when side-effects present. It was clear from patient recommendations that there was no “one-size fits all” in mode of delivery for a HNL educational resource, with individuals expressing different learning preferences.

## Conclusion

Due to the high prevalence and chronicity of HNL, self-management is a fundamental component in the overall management of HNL after completion of HNC treatment. Insights gained from the patient perspective provide an important “road map” to consider during the development of tailored HNL self-management interventions to promote engagement and adherence. Patients desire clear, factual and brief information early in cancer trajectory to elicit an understanding of HNL, with repeated/additional information at various time points in their cancer pathway. In the absence of such vital information, patients often misrepresent HNL for recurrence causing distress. When HNL is evident, patients advocate for tailored instructional information from specialists, lacking medical jargon, delivered in a mode that meets their learning style, with an initial need for reassurance as they develop skills for self-management. Having a range of educational resources is pivotal to improving confidence, motivation and adherence to HNL self-management techniques.

## Figures and Tables

**Table:**
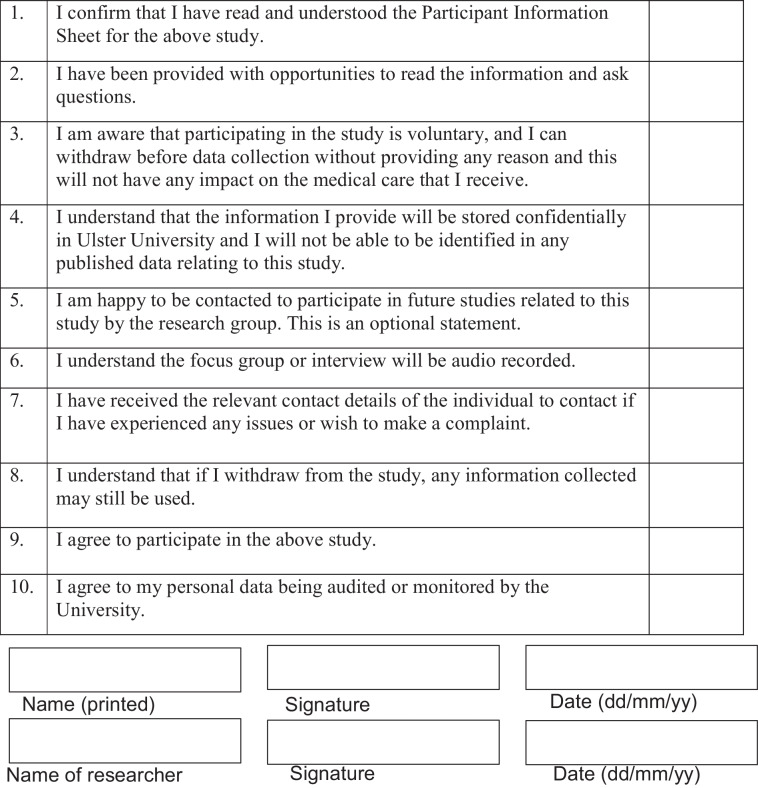
Please tick below in each box to confirm you have read and agree with the following:

## Data Availability

No datasets were generated or analysed during the current study.

## Appendix 1: Phase 1—Topic guide for interview—patient

*Research title: Planning, developing and testing a tailored self-management lymphoedema intervention for patients who have completed treatment for head and neck cancer to improve health-related quality of life.*

Every effort will be made to ensure all participants are put at ease and the topic of head and neck lymphoedema following treatment will be introduced.

Introduction

- In your own words can you explain what is your understanding of HNL? (Probe: before diagnosis and after)
- Can you give me a brief history of your HNL from when it started to present?

Head and neck lymphoedema challenges following treatment

- Describe any challenges relating to lymphoedema you may have experienced following your treatment? (Probe: home, socially, work)?
- How do these changes and challenges impact on your experience of range of motion function, swallowing, eating, body image and socialising with others? (Probe: home, socially, work)?
  - Probe: what about every day activities etc., are you still able to carry these out as normal?
- Has your ability to perform daily tasks and social interaction changed over time, since you finished treatment?
  - What/who has helped your treatment lymphoedema effects? (Probe: role of family member or carer?
- Describe how or if any changes to your appearance or functional ability has impacted on any relationships (friends/family/partner/work colleagues)?
- Has lymphoedema had an impact on your partner/family member?
- Is there anything that your friends/family/partner/colleagues done that has been improved or facilitated your lymphoedema symptoms?
- Have you experienced anything that has improved or facilitated your lymphoedema symptoms? Do people do anything that is good or helps?

Facilitators/barriers

- What techniques or advice from your specialist therapist have you found beneficial? (Probe: compression, MLD, SLD, skin care, exercises).
- Has there been a specific technique that has worked for you to cope with post-treatment lymphoedema?
- Did you know what lymphoedema was before diagnosis? (Probe: how? Was this during treatment?)
- Would a specific time when HNL was discussed have been helpful?
- What information would you have liked/felt useful?
- Are there any reasons that prevent you from carrying out self-management at home?
- What would encourage you to keep going to maintain self-management?

Coping

- Is there anything that has helped you cope with your lymphoedema symptoms? (Probe: physically, emotionally, psychologically, socially)?
- What do you do that helps you?
- Explore strategies used to improve lymphoedema self-management—(Probes: compression, MLD, sleeping techniques, peer support, exercises, advice, problem solving)?
- What strategies have you found helpful?
  - Anything that has been unhelpful?
- Explore key barriers to self-management of lymphoedema at home following treatment.

Support

- Have you received any help or support for your lymphoedema? (Probes: from family/friends/HCPs)?
  - Who has been helpful? What has been helpful? What has not been useful to you?
- What support would you like, or do you think would be beneficial?

Intervention

- What information do you think should be provided about HNL?
- What information would you like? (Probe: when would this be best?)
- Who is best placed to provide other patients with information on how best to manage post-treatment head and neck lymphoedema difficulties? (Probe: HCPs, other patients, relatives)?
- What is the best way for patients to access/get this information and support? (Probes: leaflet, online, workshop/health event, during follow-up clinic from HCPs)?
- What should it look like? The format? Leaflet? Online? Workshop/health event?
- If digital what format? (Pdf, booklet, video)
- If a video was used would you prefer real-life characters or animation to represent HNL?
- Should it be for patients/family/HCPs?
- When would be a good time for you to hear about this support? Before/during/after treatment? (Can you explain this/why do you think this would be helpful at this time?).

Consent Form—Phase 1.

*Research title: Planning, developing and testing a tailored self-management lymphoedema intervention for patients who have completed treatment for head and neck cancer to improve health-related quality of life.*

**Researcher:** Lauren Mullan.
